# Regular Organic Solar Cells with Efficiency over 10% and Promoted Stability by Ligand‐ and Thermal Annealing‐Free Al‐Doped ZnO Cathode Interlayer

**DOI:** 10.1002/advs.201700053

**Published:** 2017-04-21

**Authors:** Xiaohui Liu, Hai‐Qiao Wang, Yaru Li, Zhenzhen Gui, Shuaiqiang Ming, Khurram Usman, Wenjun Zhang, Junfeng Fang

**Affiliations:** ^1^ Key Laboratory of Graphene Technologies and Applications of Zhejiang Province Ningbo Institute of Materials Technology and Engineering Chinese Academy of Sciences Ningbo 315201 China; ^2^ University of Chinese Academy of Sciences Beijing 100049 China

**Keywords:** Al‐doped ZnO, cathode interlayer, high performance, regular organic solar cells, stability

## Abstract

Landmark power conversion efficiency (PCE) over 10% has been accomplished in the past year for single‐junction organic solar cell (OSCs), suggesting a promising potential application of this technology. However, most of the high efficient OSCs are based on inverted configuration. Regular structure OSCs with both high efficiency and good stability are still rarely reported to date. In this work, by utilizing a new designed ligand‐free and non‐thermal‐annealing‐treated Al‐doped ZnO cathode interlayer, high efficiency and greatly improved stability are simultaneously realized in regular OSCs. The highest PCE of 10.14% is accomplished for single‐junction regular OSCs with active blend of poly [[2,6′‐4,8‐di(5‐ethylhexylthienyl)benzo[1,2‐*b*;3,3‐*b*]dithiophene][3‐fluoro‐2[(2‐ethylhexyl)carbonyl]thieno [3,4‐*b*]thiophenediyl]] (PTB7‐Th):[6,6]‐phenyl C_71_‐butyric acid methyl ester (PC_71_BM). Excellent device stability is confirmed as well, by keeping 90% of its initial PCE value after 135 d in N_2_, and 80% of its initial PCE value after 15 d in ambient air, respectively. Furthermore, the applicability of the designed interlayer in regular OSCs is demonstrated by other active blend systems, including the nonfullerene material. This work highlights that high efficiency and good stability can be realized simultaneously in regular OSCs as well, and will provide referential strategy and methodology for this target.

## 1 Introduction

Based on efficient active blend systems, such as thieno[3,4‐*b*] thiophene/benzodithiophene (PTB7):[6,6]‐phenyl C_71_‐butyric acid methyl ester (PC_71_BM),^1^, ^2^, ^3^ poly [[2,6′‐4,8‐di(5‐ethylhexylthienyl)benzo[1,2‐*b*;3,3‐*b*] dithiophene] [3‐fluoro‐2[(2‐ethylhexyl)carbonyl]thieno[3,4‐*b*]thiophenediyl]] (PTB7‐Th):PC_71_BM,^4^, ^5^, ^6^, ^7^ poly[4,8‐bis[5‐(octylthio)‐thiophene‐2‐yl]benzo[1,2‐b:4,5‐b′]dithiophene‐2,6‐diyl‐*alt*‐2‐ethylhexyl3‐fluorothieno[3,4‐b]thiophene‐2‐carboxylate] (PBDT‐TS1):PC_71_BM,^8^, ^9^ poly[(5,6‐difluoro‐2,1,3‐benzothiadiazol‐4,7‐diyl)‐*alt*‐(3,3″′‐di(2‐octyldodecyl)‐2,2′;5′,2″;5″,2″′‐quaterthiophen‐5,5″′‐diyl)] (PffBT4T‐2OD):PC_71_BM,^10^ and the very recently reported non‐fullerene blend systems,^11^, ^12^, ^13^, ^14^, ^15^ organic solar cells (OSCs) have achieved great progresses, with the state‐of‐the‐art power conversion efficiency (PCE) over 10% for single junction solar cells.^16^, ^17^, ^18^, ^19^ However, almost all of those reported high performance OSCs with PCE above 10%, were focused on devices with inverted configuration. And very few studies reporting high performance OSCs with regular configuration have been presented.

With naphthalene diimide‐based conjugated polymer zwitterions as cathode interlayer (CIL), Emrick and co‐workers successfully prepared the regular structure OSCs with PCE up to 10.19% based on PBDTT‐TT:PC_71_BM blend.^20^ Peng and co‐workers gained PCE of 10.10% in regular PTB7:PC_71_BM solar cell with a star‐shaped small molecular material as the CIL.^21^ At the same time, Huang and his co‐workers accomplished PCEs of 9.70% and 10.11% in regular devices, with PNDIT‐F3N‐Br as CIL, PBT7‐Th:PC_71_BM and PffBT4T‐OD:PC_71_BM as active blends, respectively.^22^ In 2015, with PTB7:PC_71_BM active blend but Al‐doped ZnO (AZO) CIL for the first time, Ackermann and co‐workers investigated systematically the opportunity to achieve efficient colored OSCs. The best PCE of 7.59% with an open‐circuit voltage (*V*_OC_) of 0.74 V, a short‐circuit current density (*J*_SC_) of 17.1 mA cm^−2^, and a fill factor (FF) of 60% was achieved.^23^ However, for that AZO nanocrystal CIL preparation, ethanolamine (EA) was utilized as stabilizer and thermal annealing has to be applied to remove the EA in film.

Unfortunately, most of these interlayers are either obtained from complex synthesis and purification, or prepared through thermal annealing, and or formed by energy‐consuming evaporation. And moreover, metal oxide semiconductor as CIL in regular OSCs with PCE exceeding 10% have not been reported. The main reason for the very limited studies of efficient regular OSCs is that, on one hand the regular OSCs have usually provided lower device performance than the relative inverted devices with the same blend system.^24^, ^25^ On the other hand, regular OSCs tend to show disadvantages in device stability (lifetime) than the inverted devices.^26^, ^27^ Because the top low‐work‐function cathode metal or the very thin interlayer in regular device tends to get degraded by exposing to moisture and oxygen in air atmosphere.^28^ However, we consider further study and development of regular OSCs are necessary and important to fundamentally understand and improve this technology. Especially the achievement of high efficient and stable regular OSCs will be significant and beneficial for the OSCs application.^29^, ^30^

To overcome the above disadvantages of regular OSCs, the top cathode interlayer must lower the energy barrier and form an ohmic contact at the cathode. It should also effectively prevent the diffusion of the top evaporating metal into the active layer and protect the active layer from degradation caused by moisture and oxygen in air.^31^ In our previous work, it has been demonstrated that nanocrystal AZO layer can act as an efficient electron extracting layer in inverted OSCs, based on the model blend system PTB7‐Th:PC_71_BM.^32^ More importantly, we found that those tiny nanocrystals possess the ability to form compact and uniform film, and present unique contacting properties to both the electrode and active layer. This has been confirmed by the obtained excellent device parameters and suggests possible potential of AZO as top CIL to achieve high efficient regular OSCs. However, before the application of the AZO as CIL in regular OSCs, two more issues must be resolved, eliminating the thermal annealing of AZO CIL and using suitable solvent for deposition on top of the PTB7‐Th:PC_71_BM, which otherwise lower the device performance.^23^, ^33^, ^34^

To achieve our target, we synthesized new AZO nanocrystals with a different method and no ligand was used anymore in the synthesis. With our ligand‐free AZO CIL, the thermal annealing treatment is not necessary in regular PTB7‐Th:PC_71_BM solar cell, which otherwise will damage the underlying active layer as its deficiency in thermal tolerance.^33^ On the other hand, we used a new solvent 2,2,2‐trifluoroethanol (TFE) to disperse the AZO nanocrystals. This commonly used solvent TFE can protect well the morphology of the underlying active layer, and hence prevent the degradation of the active layer introduced by the mutual soluble effect of the solvents. Maximum PCE of 10.14% was obtained under AM 1.5G irradiation (100 mW cm^−2^). Excellent stability both in N_2_ and ambient atmosphere was also confirmed for the regular devices. To the best of our knowledge, this performance is among the highest efficiency of single junction regular OSCs simultaneously with good stability. Moreover, high performance parameters have also been confirmed for regular OSCs with PTB7:PC_71_BM and PffBT4T‐2OD:PC_71_BM as well as the nonfullerene active layers in our study.

## 2 Results and Discussion

### 2.1 Synthesis and Characterization of AZO Nanocrystals

Different from our previous procedure, ligand like EA was not used anymore in the synthesis, with the purpose to avoid the thermal annealing treatment for the AZO CIL on top of the active layer.^23^, ^35^ The as‐synthesized AZO nanocrystals were collected by centrifugation and finally dispersed in a commonly used solvent TFE, instead of the previously used solvent (ethanol:EA). This can effectively prevent the morphology degradation of active layer due to mutual soluble effect of solvents when the AZO CIL is deposited on top of the active layer.

The morphological features of the AZO nanocrystals were studied by transmission electron microscopy (TEM). As shown in Figure S1 in the Supporting Information, the average size of AZO nanocrystals was estimated to be about 4.9 ± 0.5 nm, about half the size of the results reported by Ackermann.^23^ A typical wurtzite crystal structure was determined for the obtained AZO sample by X‐ray diffraction patterns (XRD) (Figure S1b, Supporting Information).^36^ All the diffraction peaks can be indexed to the standard pattern of ZnO (PDF#36‐1451). Ultraviolet photoelectron spectroscopy (UPS) was performed to determine the work function of the AZO film on indium tin oxide (ITO) substrate, which was determined to be 3.91 eV, according to the measured photoemission cut‐off and valence band of the AZO film (Figure S2, Supporting Information). In combination with the optical bandgap of 3.39 eV derived from the UV absorption spectrum, the valence band and conduction band level were determined to be around −7.45 and −4.06 eV, respectively.

### 2.2 Device Configuration and Performance

Based on our previously EA stabilized AZO and combined with thermal annealing at 140 °C, although high performance with PCE 10.42% has been achieved for inverted PTB7‐Th:PC_71_BM solar cell.^32^ However, the regular structure PTB7‐Th:PC_71_BM solar cells presented only poor performance with PCEs of 2.20% and 1.66% based on the AZO (EA) CILs with and without thermal annealing, due to the thermal tolerance and solvent erosion problems of the active layer, introduced by deposition of this AZO solution on top of active layer (Figure S3 and Table S1, Supporting Information).

**Figure 1:**
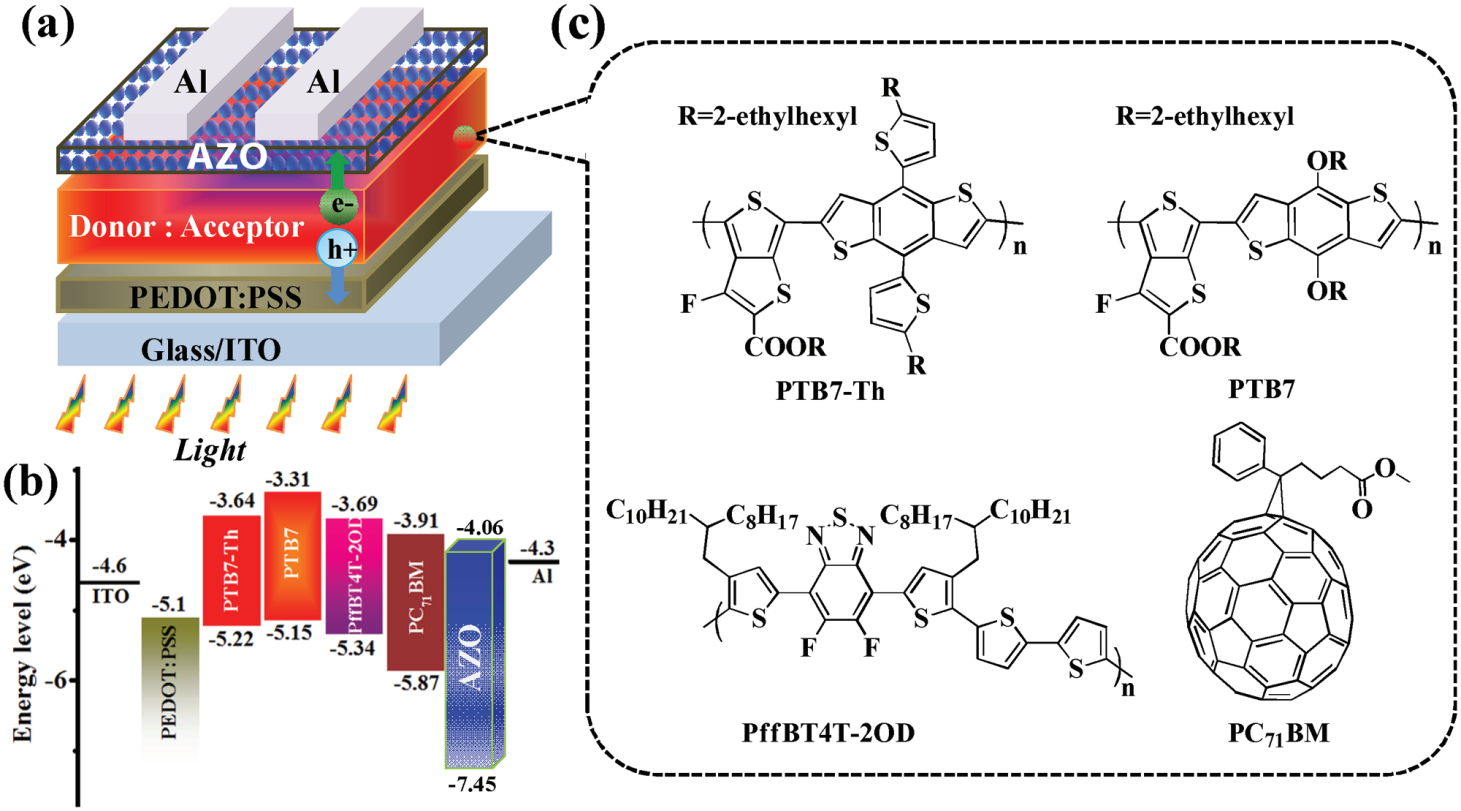
a) Configuration of regular structure OSCs fabricated in this work, ITO/PEDOT:PSS/donor:acceptor/AZO/Al. b) Corresponding energy levels of the materials used in this work. c) Molecular structures of the active layer materials: PTB7‐Th, PTB7, PffBT4T‐2OD and PC_71_BM.

However, with the ligand‐free and TFE dissolved AZO as the CIL, promising performances of regular OSCs were obtained. The regular device structure and corresponding energy levels of all materials are presented in **Figure** **1**a,b.^4^, ^37^ The lowest unoccupied molecular orbital and highest occupied molecular orbital of AZO are estimated to be −4.06 and −7.45 eV, respectively, which could effectively promote the electron transport and block the hole transport to the cathode. Figure 1c shows the chemical structures of donor polymers and acceptor used in this work.

**Figure 2:**
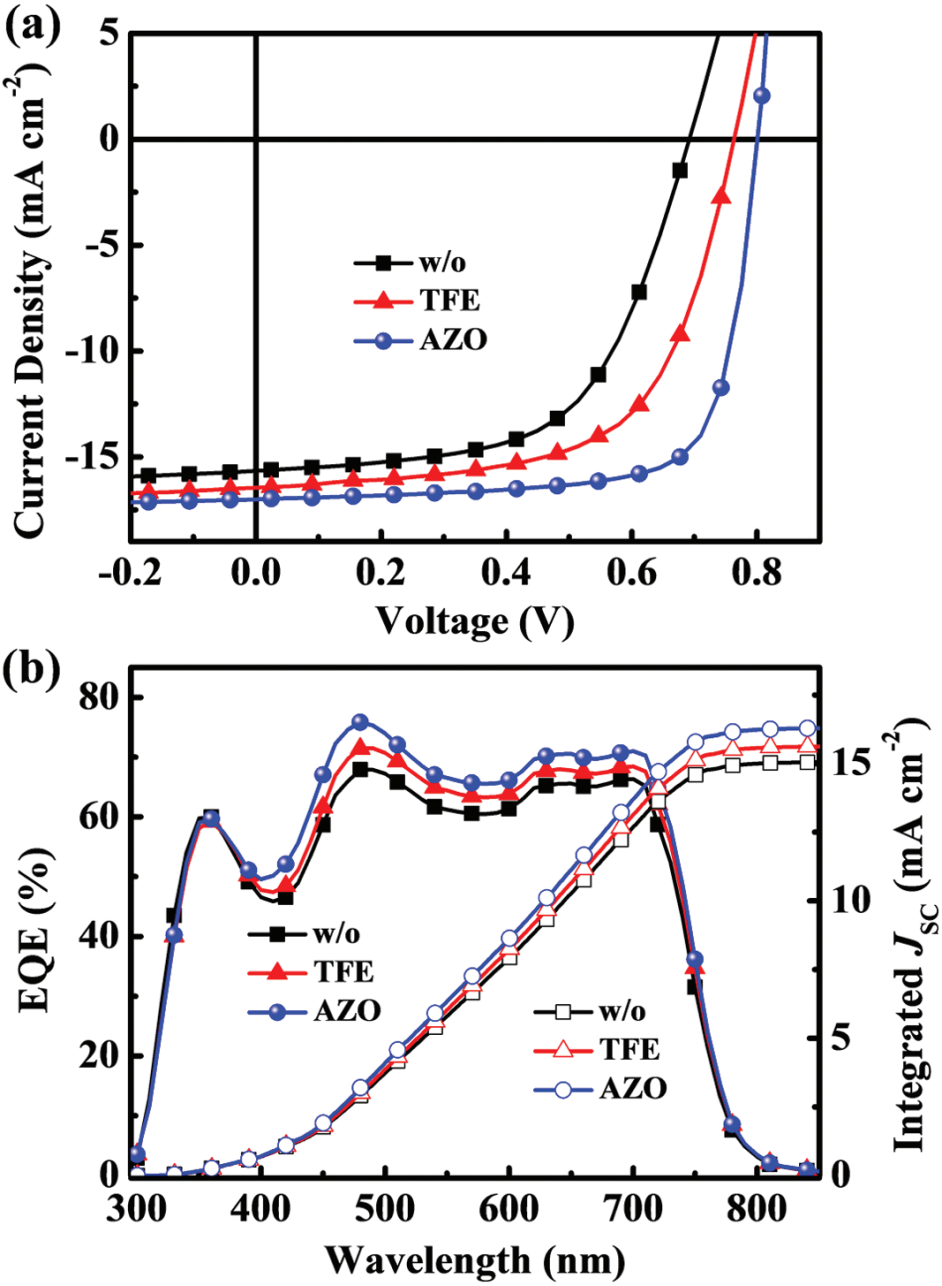
a) *J–V* characteristics measured under AM 1.5G irradiation (100 mW cm^−2^) and b) EQE curves for regular PTB7‐Th:PC_71_BM OSCs devices with different cathode interlayers (w/o, TFE, and AZO, respectively).

Since new solvent TFE was used to dissolve the AZO nanocrystals, regular OSCs with and without TFE‐treatment for the active layer were both fabricated as references, to distinguish and eliminate the influence of the TFE solvent. **Figure** **2**a shows the current density versus voltage (*J–V*) characteristics of the PTB7‐Th:PC_71_BM‐based regular devices with different CILs (w/o, TFE‐treated and AZO, respectively) under AM 1.5G illumination (100 mW cm^−2^). The untreated device, i.e., with bare Al as cathode, presented a very poor performance with an average PCE of 5.88 ± 0.38% (**Table** **1**). With the pure solvent TFE treatment of the active layer, the device showed a significant improvement, providing the best PCE of 7.81%, with a *V*_OC_ of 0.763 V, a *J*_SC_ of 16.43 mA cm^−2^, and a FF of 62.2%, indicating an obvious solvent effect on the device performance, which is similar to the effect of methanol‐treatment previously reported by Zhou et al.^38^ However, with the optimized ligand‐free and non‐annealing treated AZO interlayer, a dramatically improved performance was achieved, with a PCE of 10.14%, a *V*_OC_ of 0.801 V, a *J*_SC_ of 16.94 mA cm^−2^, and a perfect FF of 74.8% (Figure S4, Supporting Information). Compared to the results of the reference devices, it can be concluded that the contribution to the enhanced efficiency is predominantly from AZO CIL. As far as we know, this is one of the few reports about regular OSCs presenting high efficiency.^20^, ^21^, ^22^, ^37^, ^39^, ^40^ The corresponding external quantum efficiency (EQE) spectra of the regular OSCs were characterized and shown in Figure 2b. The AZO‐based device presents a substantial enhancement in EQE in the wavelength range of 385–740 nm compared with the reference devices, corresponding to a larger photocurrent density, which is in good accordance with the measured *J*_SC_.

**Table 1:** Device parameters of regular PTB7‐Th:PC_71_BM OSCs devices with different cathode interlayers (w/o, TFE, and AZO, respectively)

Cathode interlayer	*V*_OC_ [V]	*J*_SC_ [mA cm^−2^]	FF [%]	PCE [%]	*R*_S_ [Ω cm^2^]	*R*_Sh_ [Ω cm^2^]
w/o	0.692	15.63	58.9	6.38 (5.88 ± 0.38)a)	9.7	618.2
TFE	0.763	16.43	62.2	7.81 (7.78 ± 0.33)a)	7.4	602.4
AZO	0.801	16.94	74.8	10.14 (9.87 ± 0.20)a)	3.7	1638.7

^a)^ The average values of PCE were based on 16 individual devices.

### 2.3 Original Reasons for the Improvements of Regular OSCs

**Figure 3:**
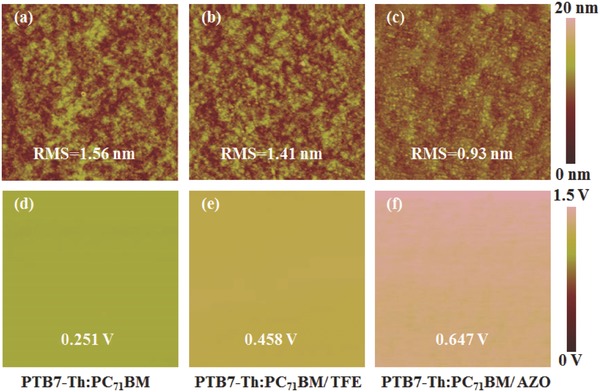
Surface topographic AFM images and surface potential images (size: 5 µm × 5 µm) of PTB7‐Th:PC_71_BM films a,d) without treatment, b,e) with TFE treatment, and c,f) with AZO interlayer, respectively. RMS roughness and surface potential values are shown in inset, respectively.

To explore the reasons for the improvement of the AZO CIL‐based regular device, the surface topographies and potentials of the relative layers were examined firstly by atomic force microscopy (AFM) equipped with standard scanning Kelvin probe microscopy (SKPM). **Figure** **3**a,b demonstrates the topographic AFM images of the active layers without and with TFE‐treatment. For both of which, similar smooth surfaces were observed. While with AZO CIL on top of the active layer, a more homogeneous surface with reduced root‐mean‐square (RMS) roughness (0.93 nm) and smaller grain size (Figure 3c) was recorded. With this closely packed AZO CIL, the direct contact between PTB7‐Th:PC_71_BM and Al electrode could be effectively avoided. The surface potential of the active layer after different treatment (w/o, TFE and AZO) was probed and the results are shown in Figure 3d–f. The PTB7‐Th:PC_71_BM with AZO CIL presents an elevated surface potential (0.647 V) than both references with or without TFE treatments (0.458 and 0.251 V, respectively). This demonstrates the enhancement of the actual built‐in potential across the device after introduce of the AZO CIL, which may have benefited the charge transport and extraction.^38^, ^41^, ^42^ These results could partly explain for the high *V*_OC_ and FF of the AZO‐based device. It is consistent with the observed lower serial resistance and higher shunt resistance (Table 1).

**Figure 4:**
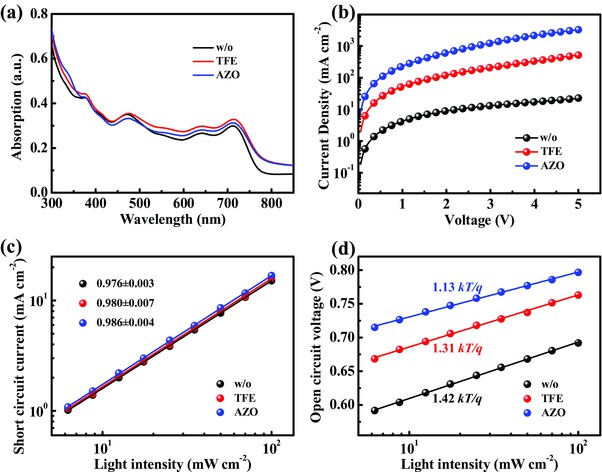
a) Absorption of PTB7‐Th:PC_71_BM films with different treatments (w/o, TFE and AZO). b) Measured *J–V* curves for electron‐only devices of ITO/Al/PTB7‐Th:PC_71_BM/CILs/Al. c) Measured *J*_SC_ of OSCs with different interlayers (w/o, TFE, AZO) plotted against light intensity on a logarithmic scale (symbols). Fitting a power law (solid lines) to these data yields α. d) Measured *V*_OC_ of OSCs with different interlayers (w/o, TFE, AZO) as a function of light intensity (symbols), together with linear fits to the data (solid lines).

**Figure** **4**a shows the absorptions of PTB7‐Th:PC_71_BM films with different treatments. The PTB7‐Th:PC_71_BM/AZO film exhibited a slightly enhanced absorption than the pure PTB7‐Th:PC_71_BM film in the whole range from 300 to 850 nm, while relatively lower absorption than the TFE‐treated active layer. This suggests the improvement in *J*_SC_ may not originate from the photoabsorption of the device.^43^ Moreover, the device electron transport ability was estimated by using space charge limited current (SCLC) measurements and fitting with the Mott–Gurney law,^41^, ^44^ based on the electron‐only devices ITO/Al/PTB7‐Th:PC_71_BM/CILs/Al. The *J–V* curves of the electron‐only devices with AZO CIL, w/o and TFE treatments are plotted in Figure 4b. Enhanced current density was observed for the device with AZO CIL compared to the w/o and TFE‐treated devices. By fitting the dark current to the SCLC model, the corresponding apparent charge carrier mobility (μ) of the AZO‐based device was calculated to be 6.14 × 10^−4^ cm^2^ V^−1^ s^−1^. It is higher than the values of the w/o and TFE‐treated reference devices (1.13 × 10^−5^ and 8.31 × 10^−5^ cm^2^ V^−1^ s^−1^, respectively), as shown in Figure S5 in the Supporting Information. This improved charge transport property could contribute to the enhancement of the device parameters such as *J*_SC_, FF, and PCE.

Meanwhile, we tested the light intensity (*P*_light_) dependences of *J*_SC_ and *V*_OC_ with gradient intensities from 6.25 to 100 mW cm^−2^ (1 sun) to investigate the charge recombination mechanism in the AZO‐based and the reference devices. The dependence of *J*_SC_ upon illumination intensity is plotted on a double log scale and can be fitted to a power law according the relation: *J*_SC_ ∝ (*P*_light_)*^α^*, where the value of the exponential factor (α) suggests the strength of the bimolecular recombination.^45^, ^46^ The closer α to unity means the less bimolecular recombination.^47^ As shown in Figure 4c, the resulting values of α was 0.986 for AZO‐based devices, 0.980 and 0.976 for the devices with or without TFE treatments, respectively. The three close α values approaching to unity indicated very weak bimolecular recombination for all the devices, especially for the AZO‐based OSCs. We also plotted *V*_OC_ versus the logarithm of *P*_light_ following the formula: *V*_OC_ ∝*nkT*/*q* ln(*P*_light_), where *k* is Boltzmann constant, *T* is absolute temperature, and *q* is electron charge.^45^, ^48^, ^49^ If a stronger dependence of *V*_OC_ on light intensity with a slope larger than *kT*/*q* could be observed, the monomolecular recombination induced by interfacial traps probably becomes dominant. As depicted in Figure 4d, the slope of the OSCs with AZO CIL is 1.13 *kT*/*q*, whereas the values of reference OSCs with or without TFE treatments are 1.31 *kT*/*q* and 1.42 *kT*/*q*, respectively, implying that the AZO CIL effectively reduced the trap density at the interface between the PTB7‐Th:PC_71_BM layer and Al. This suppressed charge recombination at the interface of active layer and cathode contributes to the high *J*_SC_ and FF, thus the enhancement of the device performances.

### 2.4 Stability of the Regular OSCs with AZO CIL

**Figure 5:**
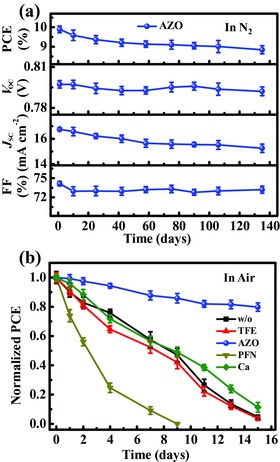
a) Measured PCE, *J*_SC_, *V*_OC_, and FF of PTB7‐Th:PC_71_BM regular OSCs with AZO CIL as a function of storage time in N_2_‐filled glovebox. b) Normalized PCEs based on different CILs (w/o, TFE, AZO, PFN, and Ca, respectively) as a function of storage time in ambient air (relative humidity: 38%–63%). The parameters were based on 16 individual devices.

As mentioned in the introduction part, stability is another crucial issue limiting the practical application of OSCs. Generally, it is believed that regular OSCs have relatively poor stability. In this work, the stability of unencapsulated regular OSCs with AZO CIL was investigated by testing the degradation of the device parameters (PCE, *V*_OC_, *J*_SC_, and FF) with time in N_2_‐filled glovebox and ambient air based on 16 individual devices (**Figure** **5**). In the N_2_‐filled glovebox, the AZO CIL device still maintained about 90% of its initial PCE (from 10.06% to 8.99%) after 135 d. Similar trend of the PCE and *J*_SC_ suggests the drop of PCE mainly caused by the gradually decreased *J*_SC_. Surprisingly, the *V*_OC_ (0.798–0.792 V) and FF (74.7%–73.6%) almost keep unchanged during the long time. The best *J–V* curves of the regular OSCs based on AZO CIL with different storage time are shown in Figure S6, and the corresponding data are listed in Table S2 in the Supporting Information.

In the ambient condition (relative humidity: 38%–63%), the AZO CIL device showed faster degradation than in the N_2_ atmosphere. The efficiency decreased to 80% of its initial value only after 15 d (Figure 5b). But this still demonstrates a decent stability for the AZO CIL device in ambient air when compared to the reference devices. As shown in Figure 5b and Table S3 in the Supporting Information, after 15 d in the ambient air, the other CIL devices (w/o, TFE and Ca) remained much lower performances (5%, 4%, and 11% of the initial PCE). And even less than 1% of the initial PCE was recorded for the PFN CIL device after 9 d. We speculated that with the addition of acetic acid (aim to dissolve the PFN in methanol), the polyfluorene PFN formed alkyl‐amine salt, which is sensitive to moisture. And once the PFN CIL treated device exposed to the ambient atmosphere, the favorable interface dipoles disappeared rapidly, resulting in the inferior device stability. The above results demonstrate that the AZO layer plays not only the role as CIL, but also as protecting layer to block the attack of moisture and oxygen, superior to the thin organic layer and air‐sensitive Ca.

### 2.5 Applicability of AZO CIL with Other Blend Systems

**Figure 6:**
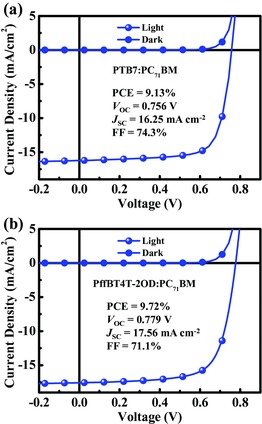
Illuminated and dark *J–V* curves of regular OSCs with AZO cathode interlayer based on the a) PTB7:PC_71_BM and b) PffBT4T‐2OD:PC_71_BM blend systems.

To examine the applicability of this AZO CIL, we also fabricated regular OSCs based on PTB7:PC_71_BM, PffBT4T‐2OD:PC_71_BM and as well as the nonfullerene active layer, poly[(2,6(4,8bis(5(2ethylhexyl)thiophen2yl)benzo[1,2b:4,5b′]dithiophene))alt(5,5(1′,3′di2thienyl5′,7′bis(2ethylhexyl)benzo[1′,2′c:4′,5′c′]dithiophene4,8dione))]:3,9bis(2methylene(3(1,1dicyanomethylene)indanone))5,5,11,11tetrakis(4hexylphenyl)dithieno[2,3d:2′,3′d′]sindaceno[1,2b:5,6b′]dithiophene) (PBDB‐T:ITIC), with the same AZO thickness (≈14 nm). The PTB7:PC_71_BM solar cell delivered an excellent performance with a high efficiency of 9.13% (**Figure** **6**a), which is comparable to the devices based on other interlayers,^42^, ^43^, ^50^ presenting huge enhancement compared to the PCE 7.59% reported by Ackermann.^23^ For the PffBT4T‐2OD:PC_71_BM blend, deposition was optimized by controlling the dropping and spin‐coating process to obtain a homogeneous morphology and optimal thickness due to the strong crystallization and temperature‐dependent aggregation behavior.^10^, ^51^ The regular PffBT4T‐2OD:PC_71_BM‐based OSCs with AZO as CIL also delivered high performance with a PCE of 9.72%, a *V*_OC_ of 0.779 V, a *J*_SC_ of 17.56 mA cm^−2^, and a FF of 71.1% (Figure 6b). Simultaneously, we tested the stability of the PTB7:PC_71_BM and PffBT4T‐2OD:PC_71_BM based OSCs in the ambient air without encapsulation. The detailed performance degradation over time is shown in Figure S7 and Table S4 in the Supporting Information. As for the popular nonfullerene PBDB‐T:ITIC system, we obtained a best PCE of 10.23%, with a *V*_OC_ of 0.909 V, a *J*_SC_ of 16.21 mA cm^−2^, and a FF of 69.4% based on the regular configuration of ITO/poly(3,4‐ethylenediox‐ythiophene):poly(styrenesulfonate) (PEDOT:PSS)/PBDB‐T:ITIC/AZO/Al, as shown in Figure S8 in the Supporting Information. These results confirm that the AZO CIL can help to achieve efficient regular OSCs in different active material systems, including the nonfullerene material.

## 3 Conclusion

In conclusion, monodisperse ligand‐free AZO nanocrystals were synthesized and dispersed well in solvent TFE. With which, AZO layer was deposited as CIL in regular PBT7‐Th:PC_71_BM solar cells. And a maximum PCE of 10.14% with a *V*_OC_ of 0.801 V, a *J*_SC_ of 16.94 mA cm^−2^, and a high FF of 74.8% was achieved. Meanwhile, excellent stability in N_2_ atmosphere and especially hugely improved stability in ambient air were confirmed for these efficient regular OSCs. This study demonstrates that the AZO CIL in regular OSCs can, on one hand elevate the interface properties like electron transporting and extracting ability at the cathode and on the other hand protect the active layer from damages by preventing moisture and oxygen. Furthermore, decent performances of regular devices with other active blend systems suggest wide applicability and promising potential of this designed CIL for achieving top level regular OSCs. Our study provides strategy and methodology for achieving high efficient and stable regular OSCs, and will promote further the understanding and development of OSCs technology.

## 4 Experimental Section

*Materials and Reagents*: Patterned ITO glass substrates (*R*_s_ ≤ 10 Ω □^−1^; transmittance ≥83%) were provided by Shenzhen huayulianhe Technology Co. Photoactive materials PTB7, PTB7‐Th, PffBT4T‐2OD, and PC_71_BM were purchased from 1‐Material Chemscitech Inc. (Canada) and American Dye Source Inc., respectively. The PBDB‐T and ITIC were obtained from Solarmer Materials Inc. (China). Aluminum nitrate nonahydrate (Al(NO_3_)_3_·9H_2_O), zinc acetate dihydrate (Zn(Ac)_2_·2H_2_O), and potassium hydroxide (KOH) were purchased from Sinopharm Chemical Reagent Co. Chlorobenzene (CB), *o*‐dichlorobenzene (DCB), 1,8‐diiodoctane (DIO) and TFE were bought from Sigma‐Aldrich. All the available chemical reagents were used as received without any further purification.

*Preparation of AZO Nanocrystals*: The AZO nanocrystals were prepared with a modified method according to the literatures. In details, Zn(Ac)_2_·2H_2_O (2.36 g) and doping source of Al(NO_3_)_3_·9H_2_O (0.08 g) were added into methanol (100 mL) in a round‐bottom flask. After the reactants completely dissolved, methanol (52 mL) solution of KOH (1.18 g) was added dropwise into the reaction solution within 12 min. The reaction mixture was stirred for 2.5 h at 60 °C. And then the products were allowed to precipitate for 2 h at room temperature. The precipitate was washed with methanol and centrifuged for collection. TFE was used to dissolve the final AZO nanocrystals.

*Device Fabrication*: The ITO‐coated glass substrates were cleaned by sequential sonication in detergent, deionized water, acetone, and isopropanol for 15 min at each step. Then the precleaned ITO substrates were treated with oxygen plasma for 10 min. A solution of PEDOT:PSS (Baytron, Clevious 4083) was spin coated onto the ITO substrates and then annealed in air at 140 °C for 20 min. Next, an ≈100 nm thick active layer was deposited by spin coating from a PTB7‐Th:PC_71_BM (1:1.5 wt%, 25 mg mL^−1^) solution in mixed solvent of CB:DIO (100:3 vol%) under 2000 rpm for 120 s. And the PTB7:PC_71_BM active blend system was processed with the same parameters. For PffBT4T‐2OD:PC_71_BM blends, an ≈300 nm film was obtained by depositing PffBT4T‐2OD:PC_71_BM (1:1.2 wt%, 19.8 mg mL^−1^) in CB:DCB:DIO (50:50:3 vol%) solution, and annealed at 80 °C for 10 min. For PBDB‐T:ITIC system, an ≈100 nm thick active layer was deposited by spin coating from a PBDB‐T:ITIC (1:1 wt%, 20 mg mL^−1^) solution in mixed solvent of CB:DIO (100:0.5 vol%), and annealed at 100 °C for 30 min. Then the AZO solution was spin coated onto the active layer at 4000 rpm for 60 s to obtain an ≈14 nm CIL. Then the devices were immediately put into the chamber for thermal evaporation and the vacuum of chamber was pumped down by two steps: 10^5^ to 10^−1^ Pa in 5 min by a mechanical pump and then 10^−1^ to 10^−4^ Pa within 10 min by a turbo pump. Keep evacuating by the turbo pump for at least 30 min. Finally, the device fabrication was completed by thermal evaporation of 100 nm Al as the electrode under a pressure less than 2 × 10^−4^ Pa. The effective device area was defined to be ≈0.06 cm^2^ controlled with a shadow mask. The electron‐only devices were made with the structure of ITO/Al/PTB7‐Th:PC_71_BM/CILs/Al.

*Characterizations*: The current density–voltage characteristics were measured inside a N_2_‐glovebox, using a Keithley 2400 sourcemeter under the illumination of AM 1.5G (100 mW cm^−2^), with a Sol3A class AAA solar simulator (Newport, model 94023A, 2 × 2 in.). The lamp was calibrated by a 2 × 2 cm^2^ monocrystalline silicon reference cell (KG5 filter) provided by Newport Corporation. The EQE measurements were performed using a Newport quantum efficiency measurement system (ORIEL IQE 200TM) with a lock‐in amplifier in air. The light intensity at each wavelength was calibrated with a standard single‐crystal Si photovoltaic cell. And the apparent charge carrier mobility (μ) was evaluated with the Mott–Gurney law, given by equation: *J* = 9ε_0_ε_r_ *μV* ^2^exp(0.89β(*V*/*L*)^0.5^)/(8*L* ^3^), where *J* stands for the current density, ε_0_ is the permittivity of free space, ε_r_ the relative permittivity of the medium (assuming that 3.4), *V* the effective voltage, *L* the thickness of the active layer, and β the field activation factor.

UV–vis spectra were recorded on a GS54T spectrophotometer (Shanghai Lengguang Technology Co., China). The film thickness was measured using a surface profiler (Veeco, Dektak 150). The phase analysis of the as‐prepared samples was performed by using a powder XRD (Bruker AXS D8 Advance, Germany) equipped with Cu Kα radiation. The morphology and size of AZO were determined by FEI Tecnai F20 TEM. X‐ray photoelectron spectroscopy (XPS) and UPS measurements were carried out using a Kratos AXIS ULTRA DALD XPS/UPS system. For XPS, survey scans to identify overall surface composition were recorded using a monochromatic Al Kα X‐ray source (1486.6 eV). UPS was performed with He I radiation at 21.2 eV from a discharge lamp operated at 20 mA, a pass energy of 5 eV, and a channel width of 25 meV. The surface potential measurements were carried out on AFM equipment with standard SKPM mode.

## Conflict of Interest

The authors declare no conflict of interest.

## Supporting information

Supplementary
