# Supplementary material for: Regular Organic Solar Cells with Efficiency over 10% and Promoted Stability by Ligand‐ and Thermal Annealing‐Free Al‐Doped ZnO Cathode Interlayer
Source: Adv Sci (Weinh). 2017 Apr 21;4(8):1700053. doi: 10.1002/advs.201700053 (PMC5566238; doi:10.1002/advs.201700053)
Supplement: Supplementary file 1 — Supplementary [file ADVS-4-na-s001.pdf]

Copyright WILEY-VCH Verlag GmbH & Co. KGaA, 69469 Weinheim, Germany, 2017.

ADVANCED SCIENCE Open Access

Supporting Information

for *Adv. Sci.,* DOI: 10.1002/advs.201700053

Regular Organic Solar Cells with Efficiency over 10% and Promoted Stability by Ligand- and Thermal Annealing-Free Al-Doped ZnO Cathode Interlayer

Xiaohui Liu, Hai-Qiao Wang, Yaru Li, Zhenzhen Gui, Shuaiqiang Ming, Khurram Usman, Wenjun Zhang, and Junfeng Fang**

Copyright WILEY-VCH Verlag GmbH & Co. KGaA, 69469 Weinheim, Germany, 2016.

Supporting Information

Regular Organic Solar Cells with Efficiency over 10% and Promoted Stability by Ligand- and Thermal Annealing-Free Al-Doped ZnO Cathode Interlayer

Xiaohui Liu, Hai-Qiao Wang, Yaru Li, Zhenzhen Gui, Shuaiqiang Ming, Khurram Usman, Wenjun Zhang, and Junfeng Fang**

X. Liu, Dr. H.-Q. Wang, Y. Li, Z. Gui, S. Ming, K. Usman, Dr.W. Zhang, Prof. J. Fang
Key Laboratory of Graphene Technologies and Applications of Zhejiang Province
Ningbo Institute of Materials Technology and Engineering
Chinese Academy of Sciences, Ningbo 315201, China
E-mail: hqwang@nimte.ac.cn, fangjf@nimte.ac.cn
X. Liu, Dr. H.-Q. Wang, K. Usman, Prof. J. Fang
University of Chinese Academy of Sciences, Beijing 100049, China

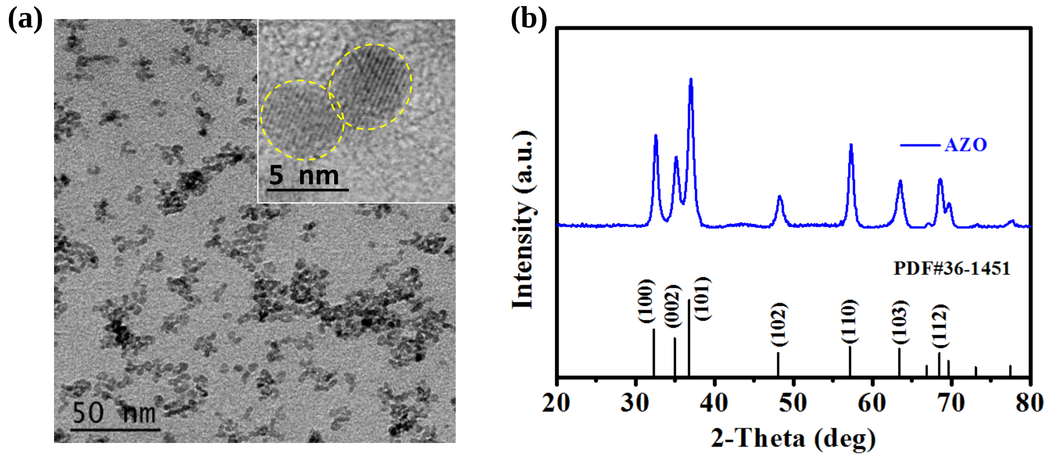

Figure S1. (a) TEM images of the AZO nanocrystals. (b) XRD patterns of AZO nanocrystals.

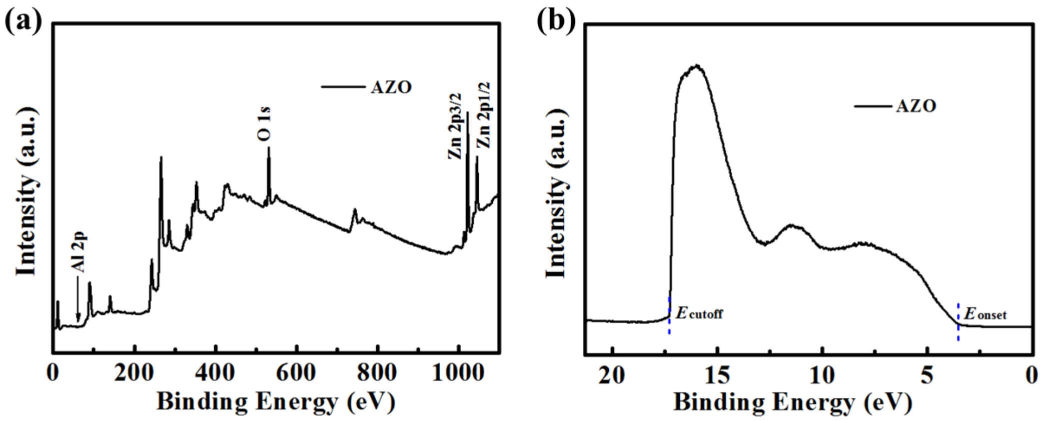

Figure S2. (a) XPS survey scan spectra of AZO film on ITO substrate. (b) UPS of AZO film on ITO substrate.

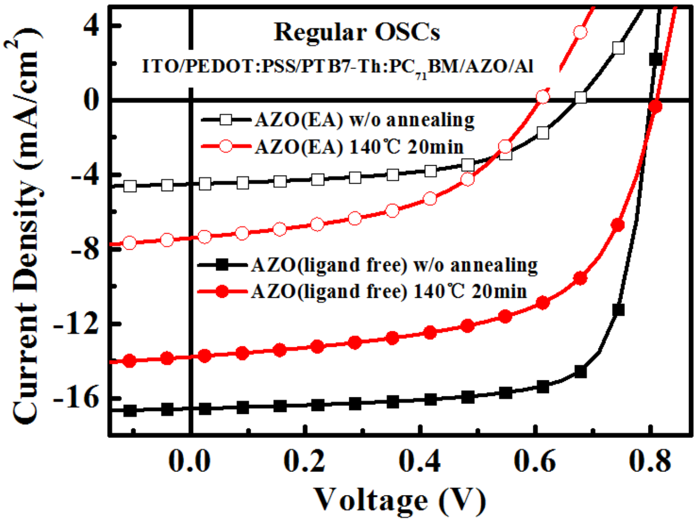

Figure S3. *J-V* characteristics of regular PTB7-Th:$PC_{71}BM$ OSCs based on EA stabilized AZO (previous work) or ligand free AZO (this work) with and without thermal annealing at 140 ^{o}C.

Table S1. Device parameters of regular PTB7-Th:$PC_{71}BM$ OSCs based on EA stabilized AZO (previous work) or ligand free AZO (this work) with and without thermal annealing at 140 ^{o}C, respectively.

CILs	V_{OC}(V)	J_{SC}(mA/cm^2)	FF(%)	PCE(%)	R_S(Ω·cm^2)	R_{Sh}(Ω·cm^2)
AZO (EA) w/o annealing	0.672	4.49	54.9	1.66	31.1	1028.8
AZO (EA) 140 ^{o}C 20min	0.608	7.39	49.1	2.20	22.6	377.6
AZO (ligand free) w/o annealing	0.800	16.83	74.5	10.03	3.8	1234.7
AZO (ligand free) 140 ^{o}C 20min	0.810	13.76	59.9	6.68	6.55	456.6

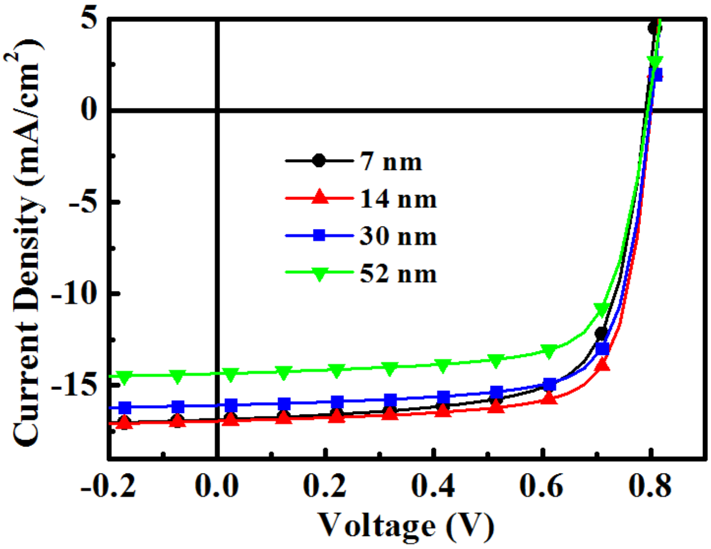

Figure S4. *J*-*V* characteristics of regular PTB7-Th:$PC_{71}BM$ OSCs based on AZO films with different thickness (7, 14, 30 and 52 nm, respectively).

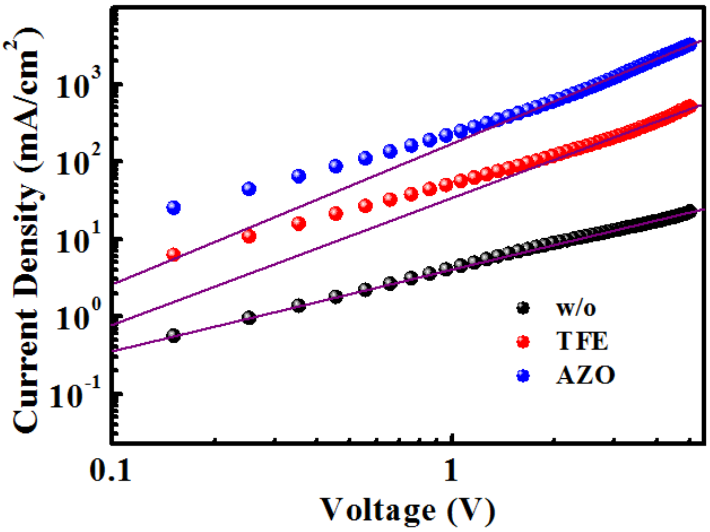

Figure S5. Current density versus bias voltage of the electron-only devices with different interlayers (w/o, TFE and AZO, respectively) in double-log scale.

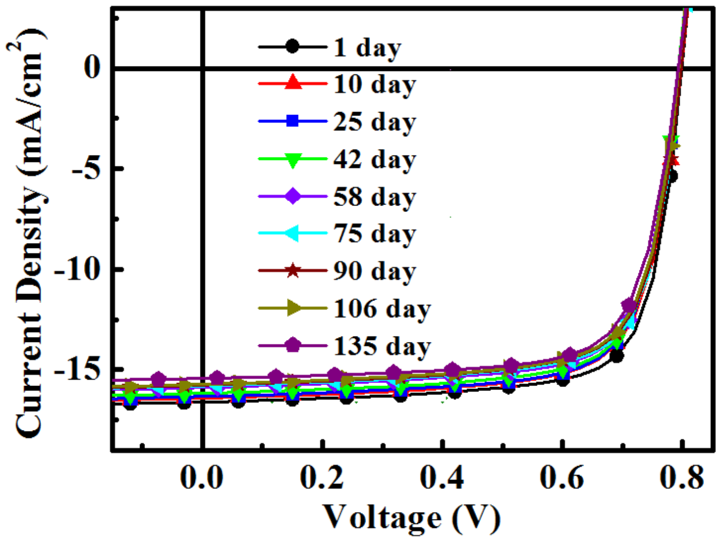

Figure S6. *J*-*V* curves of the regular PTB7-Th:$PC_{71}BM$ OSCs based on AZO interlayer, with different storage time in a N_2-filled glovebox without encapsulation.

Table S2. Device parameters of the regular PTB7-Th:$PC_{71}BM$ OSCs based on AZO interlayer, with different storage time in a N_2-filled glovebox without encapsulation.

Storage time (day)	V_{OC} (V)	J_{SC} (mA cm^{-2})	FF (%)	PCE (%)
1	0.798	16.88	74.7	10.06
10	0.797	16.68	73.1	9.72
25	0.793	16.32	73.7	9.53
42	0.793	16.16	73.7	9.44
58	0.793	15.86	73.8	9.29
75	0.796	15.78	73.8	9.27
90	0.797	15.75	73.1	9.18
106	0.793	15.72	73.3	9.14
135	0.792	15.42	73.6	8.99

Table S3. Original photovoltaic parameters of the OSCs devices based on different CILs (w/o, TFE, AZO, PFN and Ca, respectively) as a function of storage time (Initial, 9 days and 15 days, respectively) in ambient air. The parameters were based on 16 individual devices.

**CIL**	Time	V_{OC} (V)	J_{SC} (mA cm^{-2})	FF (%)	PCE (%)
**w/o**	Initial	0.692±0.005	15.63±0.31	56.9±0.5	5.92±0.24
	After 9 days	0.656±0.007	9.21±0.67	46.2±1.5	2.79±0.26
	After 15 days	0.624±0.011	1.44±0.21	31.2±1.2	0.28±0.07
**TFE**	Initial	0.761±0.004	16.23±0.42	62.2±0.7	7.68±0.26
	After 9 days	0.724±0.002	9.16±0.36	48.7±2.1	3.23±0.33
	After 15 days	0.691±0.020	1.36±0.23	30.9±1.4	0.29±0.11
**AZO**	Initial	0.798±0.003	16.72±0.34	73.6±0.6	9.83±0.18
	After 9 days	0.794±0.008	16.23±0.38	65.5±1.7	8.44±0.32
	After 15 days	0.792±0.005	15.86±0.26	62.4±1.5	7.85±0.28
**PFN**	Initial	0.791±0.004	16.34±0.35	69.1±0.7	8.94±0.21
	After 9 days	0.744±0.025	0.76±0.04	23.7±2.3	0.07±0.01
	After 15 days	/	/	/	/
**Ca**	Initial	0.789±0.003	16.12±0.28	66.3±0.5	8.43±0.15
	After 9 days	0.771±0.007	9.46±0.45	56.6±0.8	4.13±0.29
	After 15 days	0.752±0.019	2.53±0.09	49.9±0.6	0.95±0.28

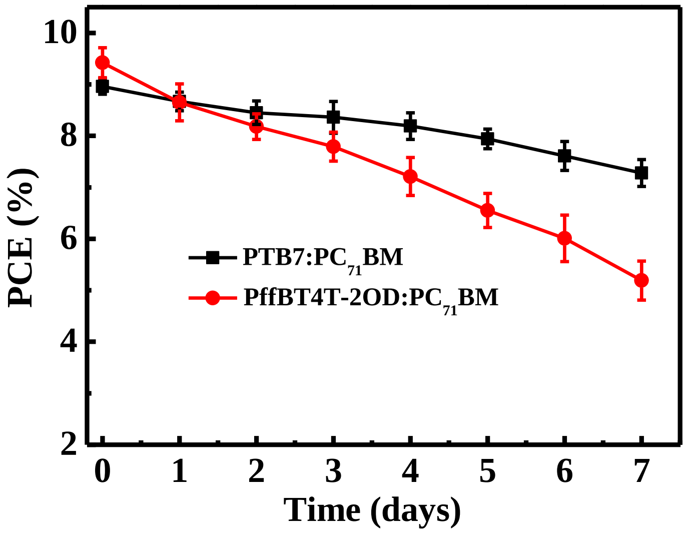

Figure S7. PCEs as a function of storage time for PTB7:$PC_{71}BM$ and Pff4TBT-2OD: $PC_{71}BM$ based OCSs with AZO CIL in ambient air. The PCEs were based on 16 individual devices.

Table S4. PCEs as a function of storage time for PTB7:$PC_{71}BM$ and Pff4TBT-2OD:$PC_{71}BM$ based OCSs with AZO CIL in ambient air. The PCEs were based on 16 individual devices.

Storage time	PCE of PTB7:$PC_{71}BM$ (%)	PCE of Pff4TBT-2OD:$PC_{71}BM$ (%)
Initial	8.96±0.15	9.42±0.29
1 day	8.67±0.18	8.65±0.36
2 days	8.45±0.23	8.18±0.25
3 days	8.36±0.31	7.79±0.28
4 days	8.19±0.26	7.21±0.37
5 days	7.94±0.19	6.55±0.33
6 days	7.61±0.28	6.01±0.45
7 days	7.28±0.26	5.19±0.38

(a)

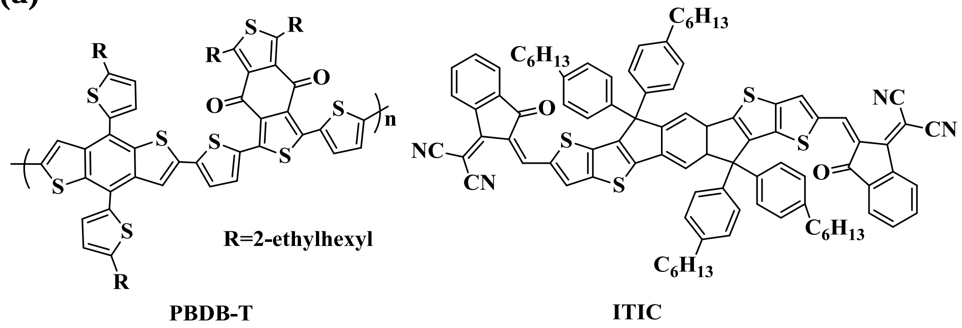

(b)

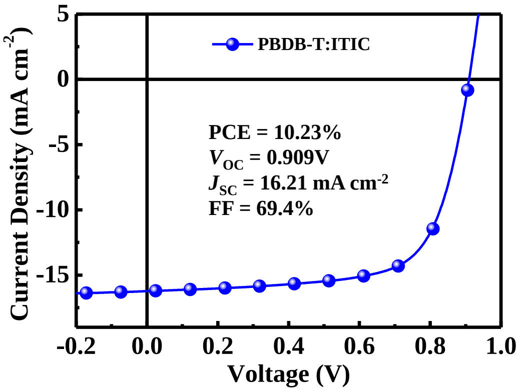

Figure S8. (a) Chemical structures of the PBDB-T and ITIC material. (b) *J*-V curves obtained with the regular device configuration of ITO/PEDOT:PSS/ PBDB-T:ITIC/AZO/Al.
